# Myocellular characteristics in rheumatoid arthritis and osteoarthritis patients

**DOI:** 10.1186/s13075-018-1557-4

**Published:** 2018-03-22

**Authors:** Sandro Manuel Mueller, David Aguayo, Daniel Aeberli, Esther Vögelin, Marco Toigo

**Affiliations:** 1 Department of Neurology, University Hospital Zurich, Zurich, Switzerland; 2 Institute of Human Movement Sciences, ETH Zurich, Zurich, Switzerland; 3 Department of Rheumatology and Clinical Immunology/Allergology, University Hospital Bern, Bern, Switzerland; 4 Department of Plastic, Reconstructive and Hand Surgery, University Hospital Bern and University of Bern, Bern, Switzerland; 5 Research and Performance Centre for Elite Athleticism, OYM, Lorzenparkstrasse 12, 6330 Cham, Switzerland; 6 Present address: Kieser Training, Zurich, Switzerland

Duijnisveld *et al.* have published an interesting study on the regenerative potential of muscle satellite cells in chronic inflammation in this journal [1]. They showed that muscle stem cell populations obtained from *M. vastus medialis* of patients with rheumatoid arthritis (RA) and osteoarthritis (OA) exhibited similar myogenic purity, viability, growth speed, differentiation, and maximum proliferative capacity. Based on these findings *in vitro*, the authors hypothesized that circulating inflammatory factors in RA negatively influence the regenerative potential of satellite cells and muscle strength *in vivo*. We aimed to verify whether these results obtained from vastus medialis muscles also apply to a muscle typically involved in the disease process of RA, namely *M. interosseus dorsalis manus 1.*

For this purpose, we obtained intraoperative muscle biopsies from the *M. interosseus dorsalis manus 1* of five RA (57.2 ± 11.1 years old) and four OA (60.7 ± 12.1 years old) patients and tested whether satellite cell numbers, myofiber sizes, and proportions were different between RA and OA patients. There was no difference in muscle fiber type distribution between RA and OA patients (Table 1). Myofiber cross-sectional area (CSA), myonuclear domains, the number of Pax7^+^ cells, and the number of proinflammatory macrophages (CD68^+^) were not different between RA and OA patients. There was a tendency for increased myonuclear number in myosin heavy chain (MyHC)-1 fibers in RA patients compared with OA patients, while there was no difference in myonuclear number in MyHC-2 fibers between the groups. MyHC-2 fiber CSAs in *M. interosseus dorsalis manus 1* were significantly larger than MyHC-1 CSAs in RA and OA patients (Table 1).

**Table 1:** *M. interosseus dorsalis manus 1* characteristics in rheumatoid arthritis and osteoarthritis patients

	Rheumatoid arthritis (*n* = 5)	Osteoarthritis (*n* = 4)
MyHC-1 (%)	74.4 ± 15.6	73.3 ± 19.8
MyHC-2A (%)	23.8 ± 13.6	23.6 ± 20.8
MyHC-2X (%)	1.8 ± 2.3	3.1 ± 3.2
CSA MyHC-1 (μm^2^)	2534 ± 714	1906 ± 773
CSA MyHC-2 (μm^2^)	4263 ± 1752*	3177 ± 1201*
MN MyHC-1	2.31 ± 0.47	1.95 ± 0.31
MN MyHC-2	3.06 ± 0.75	2.06 ± 0.66^#^
MND MyHC-1 (μm^2^)	1240 ± 389	1093 ± 323
MND MyHC-2 (μm^2^)	1523 ± 382	1354 ± 550
Pax7^+^ MyHC-1	0.038 ± 0.025	0.020 ± 0.008
Pax7^+^ MyHC-2	0.049 ± 0.075	0.028 ± 0.025
CD86^+^ MyHC-1	0.036 ± 0.019	0.034 ± 0.020
CD86^+^ MyHC-2	0.055 ± 0.025	0.038 ± 0.026

Data are shown as mean ± SD

*P < 0.05, significantly different between MyHC-1 and MyHC-2 fibers within group; ^#^*P* < 0.1, tendency for a between-group difference

*CSA* cross-sectional area, *MN* myonuclear number, *MND* myonuclear domain, *MyHC* myosin heavy chain

Our results point towards similar muscle characteristics between RA and OA patients in the highly affected *M. interosseus dorsalis manus 1*. Moreover, we found that most values for RA patients seemed to be higher when compared with OA in this preliminary dataset. Notably, there was a tendency for increased myonuclear number in MyHC-1 fibers in RA patients. Our results from a severely affected skeletal area are in line with previous studies investigating other skeletal sites. In *M. vastus medialis*, MyHC-2 CSAs were significantly larger than MyHC-1 CSAs in RA patients [2] and no significant differences in satellite cell numbers between RA and OA patients were present [3]. Based on our results from a small patient sample, the hypothesis that chronic systematic inflammation negatively influences the regenerative potential of satellite cells and myonuclei number cannot be confirmed, but it warrants further investigation.

## Abbreviations

CSA: Cross-sectional area
MyHC: Myosin heavy chain
OA: Osteoarthritis
RA: Rheumatoid arthritis
